# 15,16-Dihydrotanshinone I, a Compound of *Salvia miltiorrhiza* Bunge, Induces Apoptosis through Inducing Endoplasmic Reticular Stress in Human Prostate Carcinoma Cells

**DOI:** 10.1155/2011/865435

**Published:** 2011-01-13

**Authors:** Mao-Te Chuang, Feng-Ming Ho, Chien-Chih Wu, Shao-Yu Zhuang, Shyr-Yi Lin, Fat-Moon Suk, Yu-Chih Liang

**Affiliations:** ^1^Department of Surgery, St. Martin De Porres Hospital, Chia-Yi City 60069, Taiwan; ^2^Department of Internal Medicine, Taoyuan General Hospital, Taoyuan 33004, Taiwan; ^3^Department of Biomedical Engineering, Chung Yuan Christian University, Chung-Li 32023, Taiwan; ^4^Department of Urology, Taipei Medical University Hospital, Taipei 11031, Taiwan; ^5^School of Medical Laboratory Science and Biotechnology, College of Medical Science and Technology, Taipei Medical University, Taipei 11031, Taiwan; ^6^Department of Primary Care Medicine, Taipei Medical University Hospital, Taipei 11031, Taiwan; ^7^Division of Gastroenterology, Department of Internal Medicine, Wan Fang Hospital, Taipei Medical University, Taipei 11696, Taiwan; ^8^Traditional Herbal Medicine Research Center, Taipei Medical University Hospital, Taipei 11031, Taiwan

## Abstract

5,16-dihydrotanshinone I (DHTS) is extracted from *Salvia miltiorrhiza* Bunge (tanshen root) and was found to be the most effective compound of tanshen extracts against breast cancer cells in our previous studies. However, whether DHTS can induce apoptosis through an endoplasmic reticular (ER) stress pathway was examined herein. In this study, we found that DHTS significantly inhibited the proliferation of human prostate DU145 carcinoma cells and induced apoptosis. DHTS was able to induce ER stress as evidenced by the upregulation of glucose regulation protein 78 (GRP78/Bip) and CAAT/enhancer binding protein homologous protein/growth arrest- and DNA damage-inducible gene 153 (CHOP/GADD153), as well as increases in phosphorylated eukaryotic initiation factor 2*α* (eIF2*α*), c-jun N-terminal kinase (JNK), and X-box-binding protein 1 (XBP1) mRNA splicing forms. DHTS treatment also caused significant accumulation of polyubiquitinated proteins and hypoxia-inducible factor (HIF)-1*α*, indicating that DHTS might be a proteasome inhibitor that is known to induce ER stress or enhance apoptosis caused by the classic ER stress-dependent mechanism. Moreover, DHTS-induced apoptosis was reversed by salubrinal, an ER stress inhibitor. Results suggest that DHTS can induce apoptosis of prostate carcinoma cells via induction of ER stress and/or inhibition of proteasome activity, and may have therapeutic potential for prostate cancer patients.

## 1. Introduction

The endoplasmic reticulum (ER) has several critical functions in cells including protein synthesis, folding, and posttranslational modification, as well as the regulation of intracellular calcium homeostasis [1]. Any factors that disrupt the ER structure and function will ultimately cause defects in protein synthesis, folding, modification, and accumulation in the ER. These abnormal proteins are unfolded or misfolded proteins, which can all challenge the function of the ER-Golgi network and result in ER stress [2]. Cases of intense or persistent ER stress can trigger apoptosis [3]. Besides abnormal proteins, ceramide generation induced by Ca^2+^-independent phospholipase A2 (iPLA2*β*) also participates in ER stress-induced cell apoptosis [4]. The unfolded protein response (UPR) is a cytoprotective, signaling pathways in response to ER stress in cells [5]. In mammals, three ER transmembrane proteins are activated in the UPR, including inositol requiring 1 (IRE1), protein endoplasmic-reticular-resident kinase (PERK), and ATF6, a transcription factor [6]. The PERK enhances translation of the transcription factor, ATF4, even as it represses translation of many other proteins through eukaryotic initiation factor 2*α* (eIF2*α*) in a phosphorylation-dependent manner [7]. Upon ER stress, active IRE1 results in altered splicing of X-box-binding protein 1 (XBP1) messenger (m)RNA for encoding the functional XBP1 transcription factor, and may lead to apoptosis by stimulating phosphorylation of c-Jun N-terminal kinase (JNK) and the p38 mitogen-activated protein kinase (MAPK) [8]. ATF6 is activated by S1P and S2P proteases, leading directly to the transcription of the CAAT/enhancer binding protein homologous protein/growth arrest- and DNA damage-inducible gene 153 (CHOP/GADD153) by interaction with a specific nucleotide-binding sequence [9]. These pathways eventually activate transcription of ER chaperones, such as glucose regulation protein 78 (GRP78/Bip) and proteins involved in ER-associated degradation (ERAD), which serve to restore ER homeostasis and protect cells by eliminating ER stress [10]. The ERAD system is responsible for transferring misfolded proteins from the ER lumen to the cytosol, where they are ubiquitinated and degraded by proteasomes. Proteasome inhibitors, such as bortezomib, prevent misfolded protein degradation, block the ERAD system, and subsequently result in induction of ER stress- and ER-dependent apoptosis [11]. 

*Salvia miltiorrhiza *Bunge (tan shen root) is a well-known plant used in traditional Chinese medicine to treat various entities, such as cardiovascular disease, angina pectoris, hyperlipidemia, and acute ischemic stroke [12, 13]. Tan shen extracts contain several constituents including water-soluble phenolic acids and lipophilic tanshinones [14]. Recently, other studies and our own found that extracts of tan shen exhibit significant antitumor activity by different mechanisms in various types of tumor cells. We previously showed that DHTS markedly inhibited the proliferation of breast cancer cells through induction of G1-phase arrest and increased loss of the mitochondrial membrane potential and cytochrome c release [15]. Moreover, the inhibitory activity was ranked as follows: DHTS > tanshinone I > cryptotanshinone I. Tanshinone I was also shown to induce cancer cell apoptosis in human myeloid leukemia cells [16] and human nonsmall cell lung cancer [17] whereas tanshinone IIA induced apoptosis in human HeLa [18] and rat glioma cells [19]. Although various mechanisms were proposed to explain the antitumor effects of the different tan shen constituents, such as inactivation of the PI3K/Akt/survivin signaling pathways [16], reductions of interleukin (IL)-8, Ras-mitogen-activated protein kinase, Rac1 [17], interference with microtubule assembly [18], and inhibition of constitutive STAT3 activation [19], this issue has not been convincingly clarified.

In the present study, we show that DHTS is able to potently induce ER stress in prostate carcinoma cells, as indicated by elevated levels of GRP78/Bip and CHOP/GADD153, leading to apoptosis. Moreover, DHTS caused the accumulation of polyubiquitinated proteins and HIF-1*α*, indicating that DHTS might be a proteasome inhibitor which produces ER stress or enhanced apoptosis caused by the classic ER stress-dependent mechanism.

## 2. Materials and Methods

### 2.1. Materials

DHTS was purchased from Xi'an Honson Biotechnology (Xi'an, China). The purity was about 95% according to a high-performance liquid chromatographic (HPLC) analysis.

### 2.2. Cell Culture

The human prostate carcinoma cell line, DU145 (BCRC 60348), was obtained from the Food Industry Research and Development Institute (Hsinchu, Taiwan) and cultured in 90% minimum essential medium (MEM) containing 10% heat-inactivated fetal bovine serum (FBS; Invitrogen Taiwan, Taipei, Taiwan). Cells were plated in 6-cm dishes at 5 × 10^6^ cells per dish except the MTT assay, and allowed to grow for 24 h.

### 2.3. 3-(4,5-Dimethyl-2-Thiazolyl)-2,5-Diphenyl-2H-Tetra-zolium Bromide (MTT) Assay

Cells (1 × 10^4^ cells/mL) were cultured in a 24-well plate for 24 h and then treated with DHTS for various time periods. The cell viability was determined by an MTT assay as described previously [15].

### 2.4. Western Blot Analysis

Total cellular proteins (50 ~ 75 *μ*g) were resolved by 10% or 12% sodium dodecylsulfate polyacrylamide gel electrophoresis (SDS-PAGE) and transferred onto a polyvinylidene difluoride (PVDF) membrane (Millipore, Bedford, MA) as described previously [20]. The membrane was then incubated with the following primary antibodies: anti-PARP, anti-GRP78/Bip, anti-CHOP/GADD153, antiubiquitin, anti-HIF-1*α*, antiphosphor-eIF2*α*, antiphosphor-JNK, antiphosphor-PERK, anticleaved caspase 3, anticleaved caspase 8, anticleaved caspase 9 (Cell Signaling Technology, Danvers, MA), and anti-Bcl-2 (Santa Cruz Biotechnology, Santa Cruz, CA). The membranes were subsequently incubated with an antimouse or antirabbit immunoglobulin G (IgG) secondary antibody conjugated to horseradish peroxidase (HRP; Santa Cruz Biotechnology) and visualized using enhanced chemiluminescence (ECL) kits (Santa Cruz Biotechnology).

### 2.5. Reverse-Transcription Polymerase Chain Reaction (RT-PCR)

Total RNA was isolated from cultured cells and complementary (c) DNA was prepared as previously described [21]. XBP1 cDNA was amplified by incubating 500 ng equivalents of total cDNA in 100 mM Tris-HCl buffer (pH 8.3) containing 500 mM KCl, 15 mM MgCl_2_, 0.1% gelatin, 200 *μ*M of each deoxyribonucleotide triphosphate (dNTP), and 50 units/mL Super Taq DNA polymerase with the following oligonucleotide primers: 5′-AACAGAGTAGCAGCTCAGACTGC-3′ and 5′-TCCTTCTGGGTAGACCTCTGGGAG-3′. The cDNA of glyceraldehyde-3-phosphate dehydrogenase (GAPDH) was also amplified as a control in the same method using the following primers: 5′-TGAAGGTCGGTGTGAACGGATTTGGC-3′ and 5′-CATGTAGGCCATGAGGTCCACCAC-3′. Thermal cycle conditions were as follows: 1 cycle at 94°C for 5 min, followed by 30 cycles at 94°C for 30 s, 58°C for 45 s, and 68°C for 1 min, with a final cycle at 72°C for 10 min. PCR products were analyzed on 1% agarose gels.

### 2.6. Flow Cytometric Analysis of Apoptotic Cell Death

Apoptotic cell death was analyzed by flow cytometry using the Annexin V-conjugated Alexa Fluor 488 Apoptosis Detection Kit according the manufacturer's instructions (Molecular Probes, Eugene, OR) [15].

### 2.7. Statistical Analysis

Data are presented as the mean ± the standard error (S.E.) for the indicated number of independently performed experiments. Significantly different with *P* < .05 using one-way Student's *t*-test.

## 3. Results

### 3.1. Effects of DHTS on Apoptosis of Prostate Carcinoma Cells

**Figure 1:**
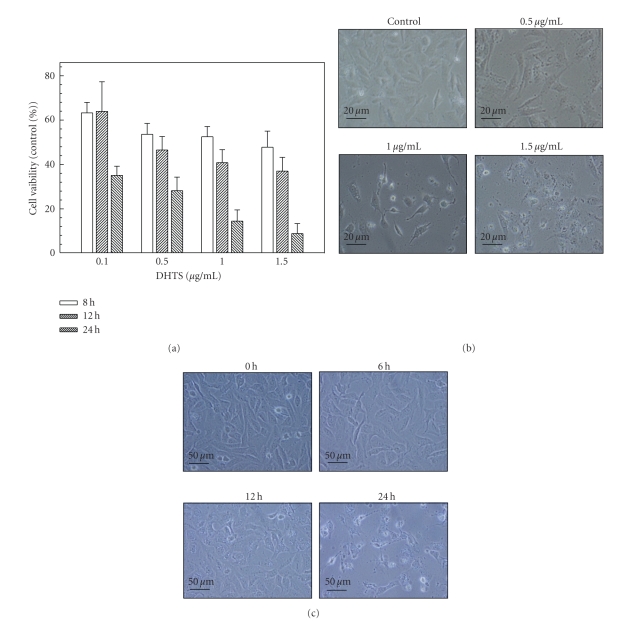
Effects of DHTS on the viability and cell morphology of human prostate carcinoma cells. (a) DU145 cells were treated with various concentrations of DHTS for 8, 12, or 24 h, and cell viability was determined by an MTT assay. Values were obtained in three independent experiments performed in triplicate and are represented as the mean ± S.E. (b) DU145 cells were treated with various concentrations of DHTS for 24 h and photographed. (c) DU145 cells were treated with 1.5 *μ*g/mL of DHTS for various time points and photographed.

**Figure 2:**
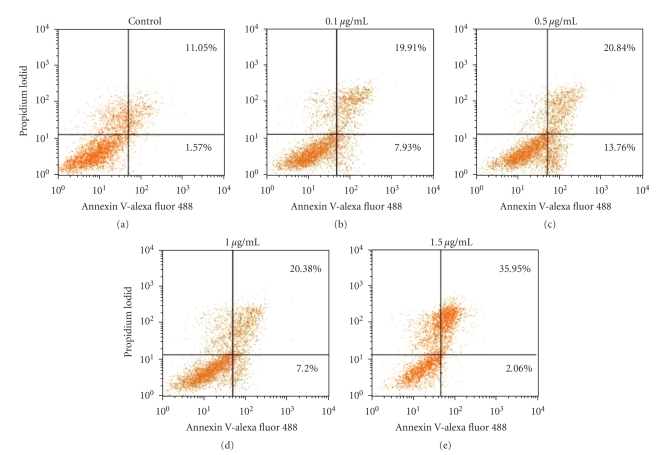
Effects of DHTS on apoptosis in human prostate carcinoma cells. DU145 cells were treated with various concentrations of DHTS for 24 h, and apoptotic cells were determined by FACS using an Annexin V-Alexa Fluor488 Apoptosis Assay Kit. The experiment was performed three times, and representative data are shown.

**Figure 3:**
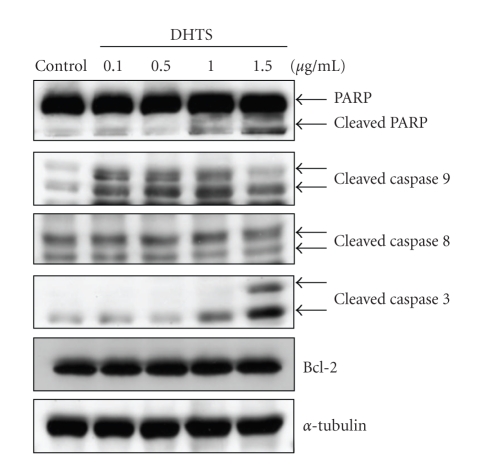
Effects of DHTS on the cleavage of caspases and PARP in human prostate carcinoma cells. DU145 cells were treated with various concentrations of DHTS for 24 h, and total cellular protein was subjected to Western blotting to detect cleavage of caspases-9, -3, and -8, PARP, and Bcl-2.

In human prostate DU145 carcinoma cells, DHTS significantly induced cell death in dose- and time-dependent manners, and showed a 64.92% and 91.18% reduction of cell viability with 0.1 *μ*g/mL and 1.5 *μ*g/mL of DHTS, respectively, at 24 h of treatment (Figure 1(a)). Using microscopic observations, cell shrinkage and rounding were found in DHTS-treated cells in dose- and time-dependent manners (Figures 1(b) and 1(c)). Cell death was also characterized using flow cytometry with propidium iodide (PI) and Annexin V-Alexa Fluor 488 staining. The lower right quadrant of the FACS histogram represents early apoptotic cells, which were stained with the green fluorescent Alexa488 dye, and the upper right quadrant of the FACS histogram represents late apoptotic cells, which were stained with both the red-green fluorescence PI and Alexa488 dyes. As shown in Figure 2, the late apoptotic cell population increased from 11.05% to 35.95% in cells treated with 1.5 *μ*g/mL DHTS. We next determined the cleavage of PARP and activation of caspases in DHTS-treated cells. After treatment with DHTS for 24 h, the cleavage of PARP and cleavage forms (active caspases) of caspases 3 and 9 were found in DHTS-treated cells in a dose-dependent manner (Figure 3). However, neither Bcl-2 expression nor the cleaved form of caspase 8 changed in DHTS-treated cells. These results suggest that DHTS induced cell death through an apoptotic pathway in prostate carcinoma cells.

### 3.2. Effects of DHTS on the Induction of ER Stress

**Figure 4:**
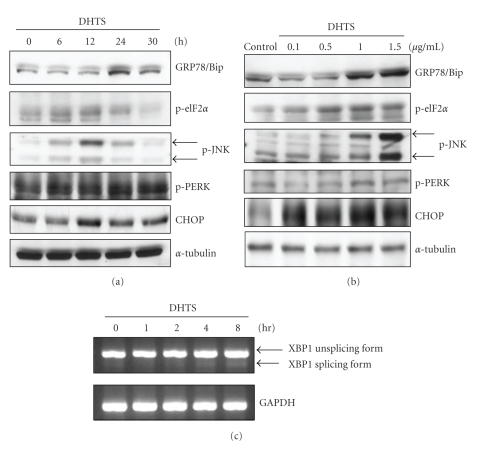
Effects of DHTS on the induction of ER stress-related protein expression and XBP1 mRNA splicing in human prostate carcinoma cells. (a) DU145 cells were treated with 1.5 *μ*g/mL DHTS for the indicated time periods, and (b) DU145 cells were treated with various concentrations of DHTS for 12 h (CHOP, p-eIF2*α*, p-JNK, and p-PERK) or 24 h (GRP78/Bip). Total cellular protein was subjected to Western blotting to detect the expressions of GRP78/Bip, CHOP/GADD153, phosphor-eIF2*α*, phosphor-JNK, and phosphor-PERK. (c) DU145 cells were treated with 1.5 *μ*g/mL DHTS for the indicated time periods, and total RNA was collected for detecting XBP1 mRNA expression by RT-PCR.

To examine whether DHTS causes ER stress in prostate DU145 carcinoma cells, several ER-responsive proteins and ER-specific signals were detected. We first measured the expressions of GRP78/Bip, which plays a role as gatekeeper in activating ER stress, and CHOP/GADD153, a transcription factor increased by ER stress. The Western blot analysis showed that the expressions of GRP78/Bip and CHOP/GADD153 significantly increased after DHTS treatment in dose- and time-dependent manners (Figure 4). We next detected the phosphorylation of ER-specific signals, including PERK, eIF2*α*, and JNK, which are known to be activated in response to accumulated unfolded proteins in the ER lumen. As shown in Figure 4, DHTS indeed induced the phosphorylation of PERK, its substrate, eIF2*α*, and JNK in dose- and time-dependent manners. The results suggested that DHTS is able to induce ER stress in prostate DU145 carcinoma cells.

### 3.3. Effects of DHTS on Inhibiting Proteasome Activity

**Figure 5:**
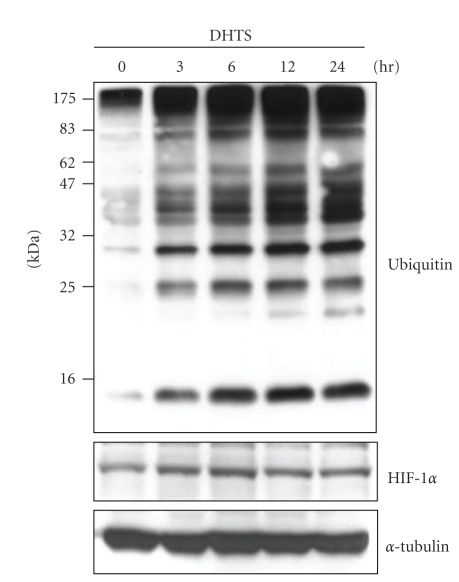
Effects of DHTS on the accumulation of polyubiquitinated proteins and HIF-1*α* protein in human prostate carcinoma cells. DU145 cells were treated with 1.5 *μ*g/mL DHTS for the indicated time periods, and total cellular protein was subjected to Western blotting to detect the expression of ubiquitinated proteins and HIF-1*α*.

To examine whether DHTS can inhibit proteasome activity, cause ER stress, block UPR, and subsequently trigger apoptosis, lysates of cells treated with DHTS were subjected to a Western blot analysis with an antibody against ubiquitin. As shown in Figure 5, polyubiquitinated proteins of various sizes were observed in DHTS-treated cells in a time-dependent manner. The rapidly degradable protein, HIF-1*α*, was also found to accumulate in DHTS-treated cells. These results suggest that proteasome activity is indeed inhibited by DHTS treatment.

### 3.4. Effects of an ER Stress Inhibitor on Reversing DHTS-Induced Apoptosis

**Figure 6:**
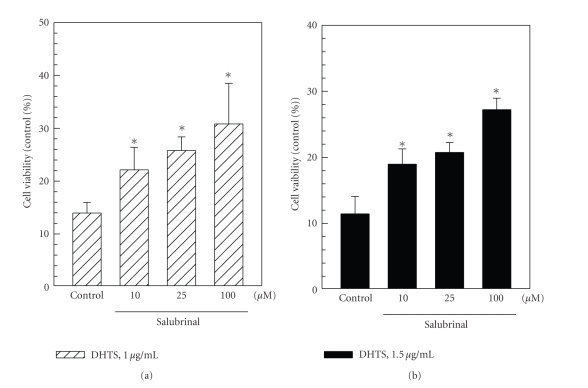
Effects of the eIF2*α* inhibitor, salubrinal, on reversing apoptosis induced by DHTS in human prostate carcinoma cells. DU145 cells were pretreated with various concentrations of salubrinal for 1 h followed by an additional treatment with 1 or 1.5 *μ*g/mL of DHTS for 24 h. Cell viability was determined by an MTT assay, and data are presented as the mean ± S.E. of three independent experiments. *P < .05, compared to individual DHTS-treated cells.

It was suggested that prolonged ER stress can cause cells to undergo apoptosis. To test whether DHTS-induced apoptosis is mediated by ER stress, salubrinal, an inhibitor of eIF2*α*, was used to block DHTS-induced ER stress. Induction of apoptosis by DHTS was significantly reduced by salubrinal (Figure 6), indicating that DHTS-induced apoptosis is partially mediated by ER stress.

## 4. Discussion

**Figure 7:**
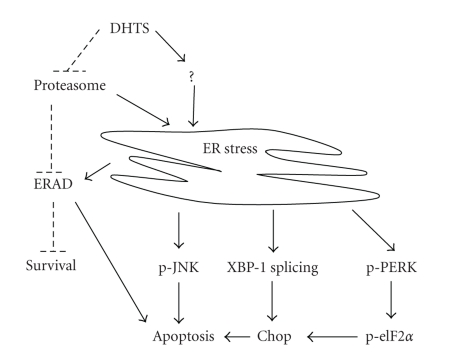
The possible mechanisms of DHTS-induced ER stress and apoptosis in human prostate carcinoma cells. First, DHTS may induce ER stress through inhibiting proteasome activity or unknown pathways. Second, ER stress induces UPR as evidenced by the upregulation of GRP78/Bip, CHOP/GADD153, and XBP1 mRNA splicing forms as well as increase of the phosphorylation of eIF2*α* and JNK. In addition, ER stress may induce ERAD, which degrades misfolded proteins mediated by proteosome. Third, prolonged ER stress causes cells to undergo apoptosis through activation of CHOP and JNK, and further promoting apoptosis through inhibition of ERAD by DHTS. Solid lines are used to indicate activating pathways, and dashed lines are used to indicate inhibiting pathways.

Tan shen is widely used in Chinese traditional medicine, and it contains many bioactive ingredients including water-soluble phenolic acids and lipophilic tanshinones. Other previous studies and our own showed that DHTS, one of the most effective of the tanshinones, was able to induce apoptosis in a number of human cancer cell lines [15–19], but the exact molecular mechanisms accounting for DHTS-induced apoptosis are not yet fully understood. In this study, we evaluated the activity of DHTS in inhibiting the growth of human prostate carcinoma cells. We found that DHTS induced apoptosis through inhibiting proteasome activity, increasing ER stress, and subsequently inducing apoptosis (Figure 7). The present study provides crucial evidence to support the involvement of ER stress in the induction of apoptosis by DHTS in human prostate carcinoma cells.

Abundant evidence demonstrated that androgens and the androgen receptor are associated with the development and progression of prostate pathogenesis [22]. In addition to androgen-independent DU145 cells, androgen-independent PC3 cells and androgen-dependent LNCaP prostate cancer cells were used to analyze the apoptotic activity of DHTS. Our results indicated that DHTS significantly inhibited both the proliferation of androgen-dependent LNCaP and androgen-independent PC3 and DU145 cells in the same manner (data not shown), suggesting that the antiproliferative effects of DHTS are not irrelevant to the androgen signal pathway.

Reactive oxygen species (ROS) are known to inhibit ER calcium pumps and ultimately result in depletion of ER calcium stores. The shortage of ER calcium causes a deterioration in the proper folding of proteins in the lumen of the ER and causes ER stress [23]. In this study, we found that DHTS significantly induced ER stress, such as upregulation of GRP78/Bip and CHOP/GADD153 protein expressions and PERK, eIF2*α*, and JNK phosphorylation (Figure 4). Other studies demonstrated that tanshinones, including DHTS, are able to induce ROS generation, and that ROS-mediated p38 MAPK activation plays a vital role in DHTS-induced apoptosis in HepG2 cells [24]. DHTS-generated ROS might contribute to the induction of ER stress in prostate carcinoma cells, but this hypothesis needs to be proven in the future.

Once ER stress occurs, cells can activate cytoprotective signaling pathways, termed the unfolded protein response (UPR), to inhibit the bulk translation via phosphorylated-eIF-2*α* and increase degradation of misfolded or aggregated proteins via proteasomes [25, 26]. Inhibition of proteasome activity was shown to enhance the antitumor activity of cisplatin and other agents that induce cell death via the classic ER stress-dependent mechanism [27]. Our results showed that DHTS might be a proteasome inhibitor due to observations of the accumulation of polyubiquitinated proteins in DHTS-treated cells (Figure 5). It is therefore possible that DHTS-induced cell apoptosis might be enhanced by its inhibition of proteasome activity, and both ER stress induction and proteasome inhibition are important in DHTS-induced apoptosis in prostate carcinoma cells.

In responses to ER stress, cells transcriptionally induced GRP78/Bip, a chaperone which assists the folding of nascent unfolded proteins and relieves ER stress [28]. However, if ER stress continues, cells express CHOP/GADD153, a transcription factor that regulates genes involved in apoptosis. Previous studies identified that CHOP/GADD153 might promote ER stress-induced cell apoptosis by downregulating Bcl-2 expression [29, 30]. In addition, DU145 prostate carcinoma cells were demonstrated to be resistant to Fas-induced apoptosis through upregulating Bcl-2 expression [31]. Cryptotanshinone, a major tanshinone, was found to sensitize DU145 prostate carcinoma cells to Fas-mediated apoptosis through suppressing Bcl-2 expression and augmenting Fas [31]. In the present study, we demonstrated that CHOP/GADD153 was induced in DHTS-treated cells (Figure 4), and inhibition of CHOP/GADD153 upstream eIF-2*α* partially reversed DHTS-induced apoptosis (Figure 6). However, the expression of Bcl-2 did not change in DHTS-treated cells, suggesting that DHTS-induced apoptosis and CHOP/GADD153-mediated apoptosis might occur in a Bcl-2-independent manner, and the underlying mechanisms of the apoptotic effects of DHTS differ from those of cryptotanshinone.

In conclusion, our study demonstrated that DHTS induces the apoptosis of human prostate carcinoma cells. The inhibitory effects of DHTS were independent of functional Bcl-2 and had no relationship with androgen responses. In this study, we first demonstrated that both ER stress and proteasome inhibition contribute to DHTS-induced apoptosis in DU145 prostate carcinoma cells. However, the detailed mechanisms through which DHTS causes ER stress and inhibits proteasome activity remain to be investigated.
